# Phenolic Compounds and Pharmacological Potential of *Lavandula angustifolia* Extracts for the Treatment of Neurodegenerative Diseases

**DOI:** 10.3390/plants14020289

**Published:** 2025-01-20

**Authors:** Olha Mykhailenko, Viktoriia Hurina, Nataliia Herbina, Yuliia Maslii, Liudas Ivanauskas, Inna Vladymyrova, Dmytro Lytkin, Zigmantas Gudžinskas, Hanna Severina, Olena Ruban, Victoriya Georgiyants

**Affiliations:** 1 Department of Pharmaceutical Chemistry, National University of Pharmacy, 61168 Kharkiv, Ukraine; viktoria.gurina2001@gmail.com (V.H.); severina.ai@ukr.net (H.S.); vgeor@nuph.edu.ua (V.G.); 2 Department of Pharmaceutical and Biological Chemistry, Pharmacognosy and Phytotherapy Group, UCL School of Pharmacy, 29-39 Brunswick Square, London WC1N 1AX, UK; 3 Department of Pharmaceutical Biology, Kiel University, 24118 Kiel, Germany; 4 Department of Drug Technology and Social Pharmacy, Lithuanian University of Health Sciences, LT-50161 Kaunas, Lithuania; nataliia.herbina@lsmu.lt (N.H.); yuliia.maslii@lsmu.lt (Y.M.); 5 Department of Analytical and Toxicological Chemistry, Lithuanian University of Health Sciences, LT-50161 Kaunas, Lithuania; liudas.ivanauskas@lsmu.lt; 6 Department of Clinical Pharmacology, Institute for Advanced Training of Pharmacy Specialists, National University of Pharmacy, 61001 Kharkiv, Ukraine; i.vladimirova@nuph.edu.ua; 7 Educational and Scientific Institute of Applied Pharmacy, National University of Pharmacy, 61000 Kharkiv, Ukraine; d.v.lytkin@gmail.com; 8 Nature Research Centre, Institute of Botany, Žaliųjų Ežerų Str. 47, 12200 Vilnius, Lithuania; 9 Department of Industrial Technology of Medicines and Cosmetics, National University of Pharmacy, 61168 Kharkiv, Ukraine; ruban_elen@ukr.net

**Keywords:** *Lavandula angustifolia*, ethnopharmacology, extraction, lyophilised extracts, neurotropic activity, phenolic compounds, antioxidant activity

## Abstract

The search for neuroprotective compounds in lavender is driven by its traditional use for brain health, with antioxidant activity serving as a key mechanism in reducing oxidative stress and supporting cognitive function. Lavender’s potential to protect neurons is based on its calming, anti-stress properties, which increase the brain’s resistance to neurodegeneration. Although lavender is not a traditional medicinal plant in Ukraine, it is increasingly recognised for its medicinal properties and is widely cultivated in the country. Lavender use in Ukraine is influenced by both global herbal practices and local medical traditions. The aim of this study was to optimise the preparation of lavender herb extracts, perform chemical profiling and evaluate their antioxidant and neuroprotective activities. The study focused on *Lavandula angustifolia* cultivated in Lviv, Ukraine. Modern analytical methods were used, including HPLC, spectrophotometry, molecular docking, lyophilisation and pharmacological testing. The selection of the optimal conditions for obtaining lavender herb extracts was determined on the basis of the results of the total yield of phenolic compounds in each extract, where it was found that the raw material–solvent ratio (1:10) in water and 50% ethanol gave the highest yield of substances; the preferred extraction time was 20 min, and the temperature was 60–70 °C, especially for water extraction. Further HPLC analysis identified marker compounds including rosmarinic acid (28.31 mg/g), chlorogenic acid (1.64 mg/g) and luteolin (0.23 mg/g) in the lyophilised ethanol extract, which were previously recognised as neuroprotective markers by molecular docking. The water extract showed higher antioxidant (total 50.85 mg/g) and neuroprotective activity, probably due to synergistic interactions among the components. Behavioural tests further demonstrated the neuroprotective potential of lavender herb. These results demonstrate the potential neuroprotective activity of lavender herb and open new possibilities for its use in the treatment of various neurodegenerative diseases.

## 1. Introduction

Antioxidant activity can be considered both a marker and an essential component of activities that improve brain function, particularly in the prevention of neurodegenerative diseases [1]. Therefore, the search for compounds in lavender (*Lavandula*) is relevant, especially given its traditional use for brain health, relaxation and stress reduction [2].

*Lavandula angustifolia* Mill. is a globally renowned plant known for its pleasant aroma, rich chemical composition and multiple pharmacological effects. While lavender is now widely used in modern traditional medicine, its practical use began much earlier in the context of local medicinal practices.

Lavender has traditionally been used to reduce stress, improve sleep quality and reduce anxiety, all of which are closely linked to brain health. The calming effects of lavender are thought to be due in part to its antioxidant properties, which reduce oxidative stress and inflammation, both of which are detrimental to brain function [3]. The knowledge gained from the traditional medicine of various cultures has led to the increased use of lavender raw materials in modern pharmaceutical practice. A significant amount of information available in the scientific literature relates primarily to essential oil, which is the most widely used product derived from lavender [4]. Due to the limited information available on the phenolic compounds in lavender herb, the focus of the current study was on crude extracts of lavender. This study highlights the novelty and importance of analysing the chemical profile and pharmacological potential of lavender herb extracts, which have been widely used in local healing practices for many years [5].

The earliest known historical references to lavender come from Ancient Egypt, where it was used in embalming and cosmetics over 2500 years ago. Initially, lavender was valued primarily for its fragrance and used in cosmetics and massage. Over time, its pharmacological properties were recognised, and it became a medicinal plant. The ancient Greeks, for example, praised lavender for its many therapeutic properties and used it to treat insomnia, back pain, headaches, and liver and spleen disorders [2]. In particular, Greek philosopher Theophrastus mentioned lavender in his work ‘On Odours’ [6]. Traditional medicine in China, Germany, France, and many other European and Arab countries has also used lavender for its antidepressant, anti-inflammatory, sedative, antispasmodic and tonic properties [7].

Today, many countries cultivate lavender for medicinal and pharmaceutical purposes, with Bulgaria and France remaining world leaders. The popularity of lavender as a medicinal plant continues to grow, driven by the discovery of new pharmacological effects supported by evidence-based medicine. Oxidative stress is an important factor in the development of Alzheimer’s disease, Parkinson’s disease, and general cognitive ageing [1,8,9]. Lavender’s antioxidant compounds, such as polyphenols and flavonoids, can neutralise free radicals and prevent neuronal damage, making it an important element in protecting brain health [10]. These chemical compounds contribute to the wide range of pharmacological effects of lavender, which has been used for many years in local treatment in various countries. These effects include its potential neuroprotective activity [11]. Due to the military aggression in Ukraine, the incidence of nervous system disorders has increased, making research in this area particularly relevant today.

Several studies investigating the neuroprotective activity of lavender extracts have demonstrated the potential efficacy of lavender extracts in the treatment of neurodegenerative diseases [11], showing improved neurological function in rats, improved memory, and reduced depression and anxiety [12], probably due to lavender’s antioxidant properties [13]. According to the scientific literature, all clinical trials were conducted on laboratory rats, and extract samples were administered intraperitoneally. The analysis of the water extract showed a reduction in depression at a dose of 200 mg/kg or 400 mg/kg [14]. Another clinical study was conducted in ethanol extracts. The authors found that intraperitoneal injections administered for 20 consecutive days resulted in decreased blood–brain barrier permeability and improved neurological function in rats, probably due to the enhancement of endogenous antioxidant defences and inhibition of oxidative stress in the brain [11]. Ethanol extracts also improved memory and reduced anxiety and depressive behaviour in a dose-dependent manner when administered at 100 mg/kg, 200 mg/kg, and 400 mg/kg per day, 30 min before each scopolamine injection and 1 h before each behavioural test on the test day [12]. Intraperitoneal administration at 200 mg/kg and 400 mg/kg, starting 1 day after injury and then daily until day 14, resulted in improved structural and functional recovery after spinal cord injury. The neuroprotective effects of lavender have led to improvements in the contusion model of spinal cord injury in Wistar rats [13]. Thus, the above studies have confirmed the beneficial effects of lavender extracts in the treatment of neurodegenerative disorders, including depression, anxiety, and impaired memory, attention and concentration. Particular attention should be paid to the prospect of using the extracts after spinal cord injury. Lavender extracts can, therefore, be used not only in the treatment of various nervous disorders but also in the recovery of the nervous system, particularly due to their antioxidant activity.

As essential oils are often used in medicinal drug development, our work aims to confirm the neuroprotective activity of lavender herb extracts through in silico, in vitro and in vivo pharmacological tests. The ‘Quality by Design’ approach is used to create a highly efficient experimental design that will allow for the identification of precise methods, the achievement of the study objectives and the evaluation of the potential applications of lavender herb extracts.

Considering the above examples and the relevance of the research direction, the current study aims to achieve the following objectives: (a) optimise extraction to maximise the yield of bioactive compounds; (b) evaluate the chemical profiles of the extracts and their antioxidant potential in vitro; (c) investigate the potential molecular mechanisms underlying the neuroprotective activity through in silico studies to identify specific markers; (d) perform in vivo pharmacological tests to assess the feasibility of using lavender extracts for the treatment of neurodegenerative diseases.

## 2. Materials and Methods

### 2.1. The Design of the Experiment

**Figure 1:**
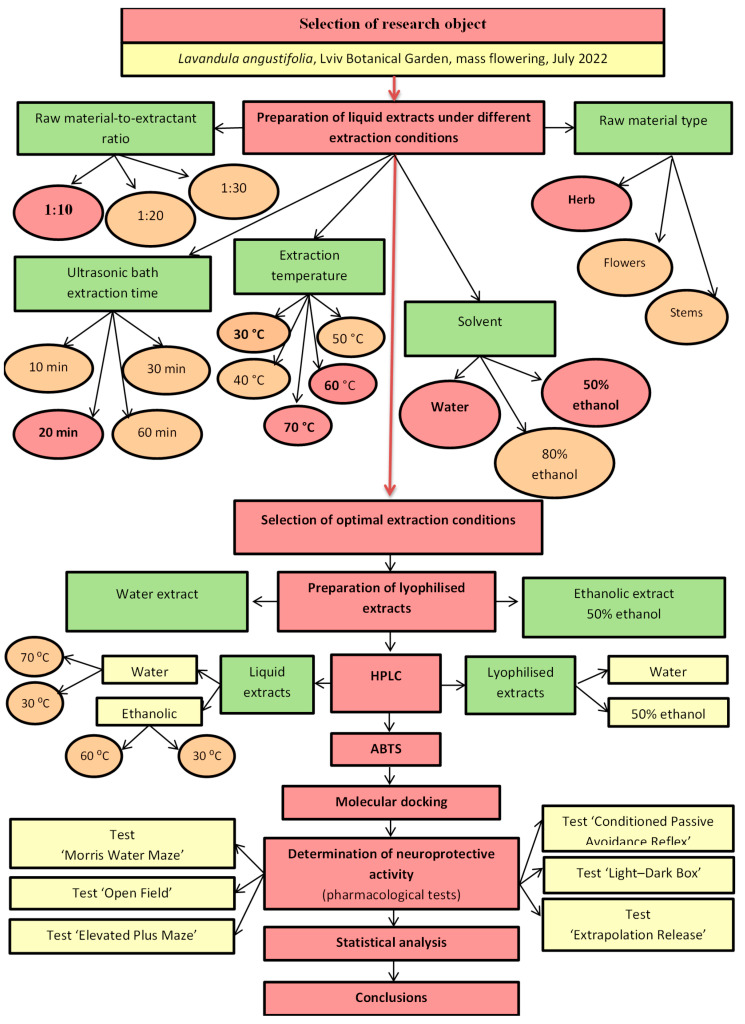
The design of the experiment.

A comprehensive experimental design (Figure 1) was developed to achieve all the objectives of this study, including the comparative analysis of lavender herb extracts, the identification of the optimal extraction conditions, the preparation of lyophilised extracts based on the results obtained and the full chemical and pharmacological analysis of the extracts.

The ‘Quality by Design’ (QbD) approach was used to design the experiment according to the ICH Q8 guideline ‘Pharmaceutical Development’ [15]. The chosen approach emphasises a clear definition of the objectives of the experiment, as well as coordination and control in each stage of the extraction technique and HPLC analysis from the selected raw materials. Based on literature data, the primary objective of the studied raw materials was identified as their potential neuroprotective value.

### 2.2. Herbal Raw Material

**Figure 2:**
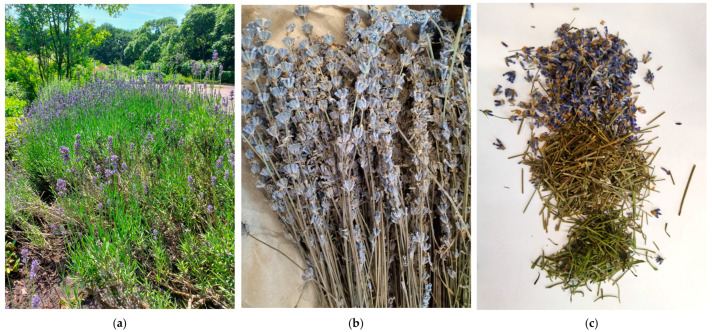
*Lavandula angustifolia* and its raw material: (**a**) general view of plants cultivated in the Lviv Botanical Garden of National University named after Ivan Franko (Lviv, Ukraine, 2022), photo by O. Mykhailenko; (**b**) general appearance of dry lavender herb; (**c**) separated flowers, leaves and stems of lavender.

Samples of the herb *Lavandula angustifolia* Mill. were collected in July 2022 during the mass flowering stage at the Lviv Botanical Garden of National University named after Ivan Franko (Lviv, Ukraine) (Figure 2a). The herbal raw material (HRM), consisting of leaves, flowers and the upper part of the stem, was air-dried in the shade at an air temperature of 30 ± 3 °C and a humidity of 25 ± 5%. The samples were then ground prior to analysis (Figure 2b,c). Stems and flowers were also analysed separately to compare their composition with that of the whole herb. The samples were identified by Dr Skybitska, and the voucher specimen (number LBC9033) was deposited in the Herbarium of National University named after Ivan Franko.

### 2.3. Standards and Chemicals

Ethanol 50% and 80% (Vilniaus Degtinė, Lithuania) and distilled water (GFL, Gesellschaft für Labortechnik GmbH, Burgwedel, Germany) were used as solvents in the extraction process. For the spectrophotometric studies, the following reagents were used: ABTS solution (98%; Sigma Aldrich, Schnelldorf, Germany), potassium peroxide solution (99%; Carl Roth, Karlsruhe, Germany), Trolox standard (98%; Fluka, Buchs, Switzerland), Folin–Ciocalteu reagent (Sigma Aldrich, Schnelldorf, Germany) and 7% sodium carbonate solution (Sigma Aldrich, Schnelldorf, Germany). Acetonitrile of HPLC grade, HPLC trifluoroacetic acid and methanol of HPLC grade were purchased from Fluka Chemie (Buchs, Switzerland). Solutions of rosmarinic acid (≥98%; Merck, Darmstadt, Germany), chlorogenic acid (≥95%; Merck, Darmstadt, Germany) and luteolin (≥95%; Merck) were used for peak detection by HPLC.

### 2.4. Extraction and Optimisation

Lavender extracts were prepared from the herb, flowers and stems in ratios of 1:10, 1:20 and 1:30 by using distilled water and 50% ethanol as solvents at varying heating temperatures from 30 °C to 70 °C with an increase of 10 °C for each extraction. The ground HRM was sonicated with 10 mL of solvent in an ultrasonic bath (WiseClean, Seoul, Republic of Korea) for 10, 20, 30 or 60 min. A centrifuge (Centurion Scientific C2, West Sussex, UK) was used to precipitate the extract to facilitate subsequent filtration. The extracts were filtered through a membrane filter (pore size of 0.45 µm), after which the samples were collected in test tubes for further analysis or stored in a freezer at −20 °C. The extracts were named as follows: LWE—Lavender Water Extract; LEE—Lavender Ethanolic Extract.

### 2.5. Total Phenolic Content Quantification

The total content of phenolic compounds was measured by the spectrophotometric method using Folin–Ciocalteu reagent [16]. For sample preparation, 0.2 mL of plant extracts was mixed with the reagent (0.2 mL), distilled water (1.8 mL) and 7% sodium carbonate (2 mL). The resulting solutions were made up to volume with water and stored in a dark place for 90 min. Absorbance was measured with a spectrophotometer Halo DB-20 (Dynamica; Livingston, UK) at a wavelength (λ_max_) of 750 nm. Gallic acid was used as the standard for the calibration curve, and the results were expressed as gallic acid equivalents (mg GAE/g). The contents of compounds in the extracts were recalculated for one gram of HRM.

### 2.6. Preparation of Lyophilised Extracts

Distilled water and 50% ethanolic extracts were prepared in a ratio of 1:10 with subsequent evaporation with Heidolph equipment (Schwabach, Germany) to crude residue and stored in a refrigerator followed by lyophilisation at −20 °C (Christ Gamma 1-16 LSC; Osterode am Harz, Germany) to obtain dry extracts. The resulting extracts were named as follows: LLWE—Lavender Lyophilised Water Extract; LLEE—Lavender Lyophilised Ethanolic Extract.

### 2.7. HPLC Analysis of Polyphenols in Extracts and Lyophilised Extracts

HPLC compound identification was performed by using a Waters e2695 chromatograph (Alliance HPLC system) equipped with a 2998 PDA detector (Waters, Milford, MA, USA). Phenolic compound analysis was performed on an ACE Super C18 column (250 mm × 4.6 mm, 3 µm) (ACT, Aberdeen, UK). Mobile phase A was 0.1% (*v*/*v*) trifluoroacetic acid in pure water (*v*/*v*), and mobile phase B was acetonitrile (*v*/*v*). A gradient solvent system separation was used: 0 min, 5% B; 8–30 min, 20% B; 30–48 min, 40% B; 48–58 min, 50% B; 58–65 min, 50% B; 65–66 min, 95% B; 66–70 min, 95% B; 70–81 min, 5% B. The flow rate was maintained at 1.000 mL/min, and the injection volume was 10 µL. The column temperature was 25 °C. Peaks in the chromatograms were identified by the comparison of retention times and on-line UV spectra with those of the standards. Quantification of the compounds was performed by using the external standard method, following established protocols from previous studies [17].

### 2.8. Analytical Method Validation

The HPLC-PDA analytical method was validated according to the guidelines of the International Conference on Harmonisation (ICH) [18]. Numerous tests were carried out to optimise the chromatographic conditions. Specificity was tested by comparing the retention times and UV spectra of the substances in the extracts with a reference compound. Identification was performed by scanning a wavelength range of 200–400 nm. For quantification, 5- to 7-point linear calibration curves (*r* > 0.999) were constructed by plotting the response of each analyte against target concentrations (ranging from 1.6 to 200.0 µg/mL). Limits of detection and quantification were calculated by using the formulae LoD = 3.3 × δ/S and LoQ = 10δ/S, respectively (δ—standard deviation of the intercept; S—slope of the calibration curves). Precision, expressed as percentage relative standard deviation (RSD, %) of peak areas, was assessed for repeatability on the day of injection and for intermediate precision over three consecutive days, with all values not exceeding the 2% threshold (Table 1). The recovery percentages of the analysed compounds fell within the acceptable range of 90–110% for the concentration levels studied, confirming the validity of the method. The quantity of metabolites was calculated by using external standard calibration within the concentration range of 0.5–100.0 µg/mL (*r*^2^ = 0.997). The study was repeated twice for each sample, and the average value was used for calculations.

### 2.9. ABTS Radical Scavenging Activity Assay

HPLC-PDA and HPLC-ABTS were performed by using a Waters Alliance 2695 UV/VIS detector (Waters Corporation, Milford, MA, USA) separation module system as previously described by Marksa et al. with some modifications [19]. The antioxidant activity was expressed as Trolox equivalent antioxidant capacity (TEAC) and was obtained from a standard calibration curve prepared from ethanolic Trolox solution at six dilutions in the range of 4.45–252.46 µg/mL; it was expressed by the equation *y* = 1.46 × 10^4^*x* + 9.52 × 10^3^, R^2^ = 0.999. TEAC corresponds to µmol of Trolox equivalent (TE), which has the same antioxidant activity as one gram of dry plant matter under the same experimental conditions (µmol Trolox/g DM). To prepare the solution, ABTS reagent (7 mM) was combined with potassium peroxide solution (2.45 mM) and stored in the dark for 12–16 h.

### 2.10. Molecular Docking

Molecular docking was performed by using the AutoDock Vina and AutoDockTools 1.5.6 programs [20]. Visualisation and analysis of the docking results obtained were performed by using Discovery Studio Visualizer. Macromolecules from the Protein Data Bank [21] were used as biotargets: •PDB ID 5NAU, 7SAD. A virtual database of candidate structures was constructed by using BIOVIADraw 2021 and saved in *.mol format. The structures were optimised by Chem3D with the MM2 molecular mechanics algorithm, saved in .pdb format and converted into *.pdbqt by using AutoDockTools-1.5.6. Discovery Studio Visualizer 2021 was used to remove solvent and native protein ligand. The prepared macromolecule was then saved in *.pdb format. In AutoDockTools-1.5.6, polar hydrogen atoms were added to the protein structure, which then was saved in *.pdbqt format. The size of the grid box and its centre were determined by the native ligand of subunit A:

AChE (PDB ID 5NAU): x = 113.6, y = 126.6, z = 181.6; size x = 34, y = 26, z = 34.

NMDAR (PDB ID 7SAD): x = 167.9, y = 174.7, z = 216.4; size x = 10, y = 10, z = 8.

Macromolecules from the Protein Data Bank were used as target proteins:

- Acetylcholinesterase (AChE) enzyme (PDB ID 5NAU) isolated from *Tetronarce californica* with a native active-site inhibitor, BPMI ((2E)-2-[(1-benzyl-4-piperidyl)methylene]-5-methoxy-indan-1-one); donepezil, a selective AChE inhibitor, was used as a reference ligand [22];
- Ionotropic NMDA glutamate receptors (PDB ID 7SAD) isolated from *Rattus norvegicus* in conformation with a selective blocker, memantin [23].

To analyse the effectiveness of the in silico assay parameters used in reproducing the experimental conformational data, native reference ligands were docked into the corresponding active sites by using the redocking procedure. The reproducibility of the binding in the active sites described in [22,23] was successfully achieved. The root-mean-square deviation (RMSD) values between the native and reference conformations were calculated by using the online resource ProFit Results and yielded values of 2.022 Å for BPMI and 1.205 Å for memantine. These results confirm the reproducibility of the experimental data and the validity of the chosen methodology.

### 2.11. Neurotropic Assay In Vivo

#### 2.11.1. Animals

Randomly bred male Wistar rats (*n* = 108), with an average weight of 190–210 g, were obtained for the pharmacological study from the Central Scientific-Research Laboratory at the Educational and Scientific Institute of Applied Pharmacy of National University of Pharmacy (Kharkiv, Ukraine). Animals were housed in standard cages under a light/dark cycle of 12:12 h in temperature-controlled rooms (22 ± 1 °C) with restricted access to rodent chow and water. All experiments were conducted in accordance with European Union Directive 2010/63/EU [24]. Animals were randomly assigned to one of three experimental groups (*n* = 6 per group) for six independent tests. Depending on the group, the animals received one of the following treatments: LLWE, LLEE, or water (control group). Samples were administered intragastrically at a dose of 300 mg/kg for five consecutive days [25,26]. A series of behavioural tests were performed at the end of the dosing period.

#### 2.11.2. Effect of Test Samples on Spatial Memory

The Morris Water Maze test was performed to assess the effects of the test samples on spatial memory and learning. The experiment took place in a circular plastic pool 120 cm in diameter (height: 55 cm; depth: 45 cm), filled with water at 26 °C. Eight plastic geometric figures of different colours and sizes (external landmarks) were placed equidistantly around the perimeter of the pool, dividing the perimeter of the pool into four segments corresponding to the cardinal points [27].

On the third day of extract administration, an initial phase was performed for all animals: the platform was placed 1.5 cm above water level. For the next two days, the animals received the extracts and solvent and were trained to locate the platform. Each animal was placed in the pool three times a day from different starting positions (facing the wall) for a duration of 60 s. Three distal positions equidistant from the platform were chosen as starting points. Animals that failed to locate the platform within 60 s were placed on the platform for 20 s. Those that successfully found the platform remained on it for 15 s. In the control experiment, the platform was submerged 1 cm below the water surface, and the water was dyed with food colouring to make it visible. A video camera recorded the sessions, and the latent time to reach the platform within a two-minute period and the percentage of time spent in the quadrant containing the platform were measured [28].

#### 2.11.3. Assessment of Behavioural Elements

The Open Field test was used to assess individual behavioural elements in rats. The apparatus consisted of a white square platform measuring 60 × 60 cm, raised on legs and illuminated by a 60 W lamp. The sides of the platform were 30 cm high, and the floor was divided into 16 identical squares, each measuring 15 × 15 cm, with 4 cm diameter holes in the centre of each square. Scoring criteria included the number of squares crossed, holes explored, instances of vertical standing, faecal pellets, urination acts and grooming behaviour observed over a 3 min period [29].

#### 2.11.4. Assessment of Animal Anxiety Levels

The Elevated Plus Maze test was used to assess the level of anxiety in the animals and determine the anxiolytic properties of the samples. The apparatus consisted of two open arms measuring 50 × 10 cm and two enclosed arms of the same dimensions, each with 10 cm high sides. The test area was illuminated by a 60 W lamp, and the maze was raised 75 cm above the floor. After a 5 min period in a darkened cage, the animal was placed in the centre of the maze, facing one of the open arms. The test lasted 5 min, during which the latency to enter the dark arm, the time spent in both the light and dark arms, and the number of transitions between arms were recorded [30].

#### 2.11.5. Assessment of Effect on Memory Formation and Reproduction

The Conditioned Passive Avoidance Reflex (CPAR) test was performed without the use of amnesic agents to investigate the effects of the substances on memory formation and retention. On the 4th day of treatment, CPAR was induced by administering an electric shock to the animals after they had been placed in the dark chamber of the apparatus. Twenty-four hours later, the presence of CPAR was assessed. Animals were observed for 3 min, during which the time taken to enter the dark chamber was recorded, along with the number of animals exhibiting a formed reflex [31].

#### 2.11.6. Assessment of Animal Behaviour with a Free Choice of Comfortable Conditions

The Light–Dark Box test was used to assess the behaviour of the animals in choosing comfort conditions. The setup consisted of an arena divided into two sections by a partition with a small opening: one section was painted white and brightly lit, while the other was dark and enclosed. Each compartment measured 50 × 30 cm, with walls 50 cm high. The rats were placed in the bright section of the arena, and the experiment lasted 5 min for each animal. The time spent in both the dark and light compartments and the number of transitions between them were recorded [32].

#### 2.11.7. Assessment of Effect on Animal Cognitive Functions

The Extrapolation Release test was used to evaluate the effect on cognitive function under acute stress in an aversive environment. The setup consisted of a transparent plastic cylinder, 50 cm high and 10 cm in diameter, immersed 2 cm in a 5 dm^3^ container filled with water (22–24 °C). Twenty minutes after the last injection of the test samples, the animals were gently placed in the cylinder headfirst, and their behaviour was observed for 3 min. The time taken for the rats to dive under the edge of the cylinder (the only escape route) was recorded, along with the percentage of rats that successfully completed the test within the 3 min time frame [33].

### 2.12. Statistical Data

Data were processed by using Microsoft Office Excel 2010 (Microsoft, Albuquerque, NM, USA) and the LabSolutions system (Shimadzu, Kyoto, Japan) to manage operational procedures and calculations. A significance level of *p* < 0.05 was set. The results of the pharmacological tests were expressed as the median [Q1; Q3]. The effects of solvent, extraction time and plant material-to-solvent ratio were estimated by using two-way permutational analysis of variance (Euclidean distance and 9999 permutations). Comparisons between study groups were made using non-parametric analysis methods, including the Mann–Whitney U test and Fisher’s *t*-test. Statistical analyses were performed by using the MS Excel 2007 Basic package and IBM SPSS Statistics 22 [34].

## 3. Results

### 3.1. Preparation and Analysis of Lavender Liquid Extracts

**Figure 3:**
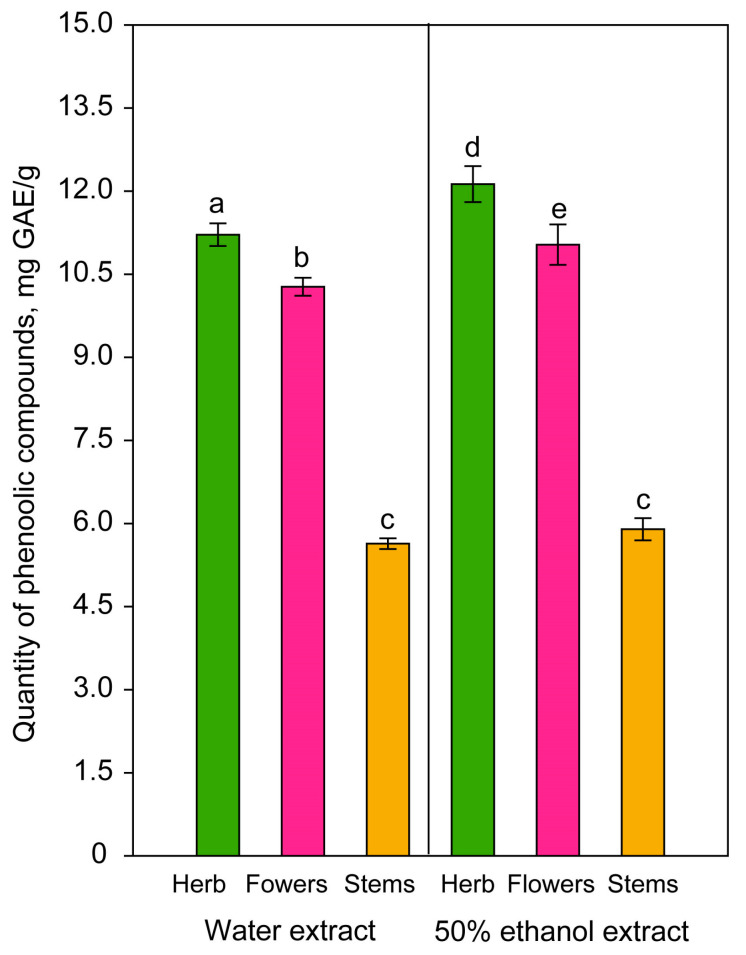
Quantity of phenolic compounds extracted from herb, flowers and stems of lavender. Whiskers represent the standard deviation. Different letters above the bars indicate significant differences (*p* < 0.05) between variants.

Different extraction conditions were used to determine the optimum technology for obtaining plant extracts, by comparing the values of the yield of phenolic compounds in the extracts. To justify the separation or non-separation of HRM and the feasibility of using lavender herb, a comparative study of the chemical composition of the inflorescences and stems was first carried out (Figure 3). The mean content of extracted phenolic compounds from lavender parts was higher when 50% ethanol was used than when water was used as solvent. However, extracts from individual parts still had lower contents than the whole aerial parts of lavender. Stems had the lowest yield of total phenolic compounds, ranging from 5.64 ± 0.10 mg GAE/g to 5.90 ± 0.20 mg GAE/g, whereas extracts from flowers had relatively high content of phenolic compounds, ranging from 10.27 ± 0.16 mg GAE/g to 11.03 ± 0.37 mg GAE/g. In comparison, a high yield of phenolic compounds was found in lavender herb extract in 50% ethanol (12.13 ± 0.32 mg GAE/g), which was significantly (*p* = 0.006) higher than in its water extract (11.21 ± 0.20 mg GAE/g). In stem extracts in both solvents, the content of phenolic compounds was significantly (*p* < 0.001) lower than in other plant parts, and the difference between solvents (*p* = 0.776) was not significant (Figure 3).

The results obtained for the total yield of phenolic compounds indicate that it is not necessary to separate the lavender herb, as the whole herb can be used, thus ensuring a comprehensive use of the raw materials.

**Figure 4:**
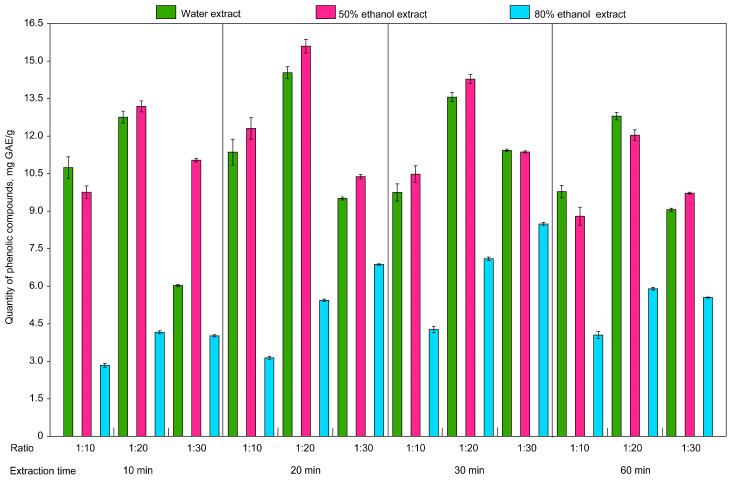
Effect of extraction time, type of extractant and ratio of HRM to extractant on total content of phenolic compounds from lavender herb. Whiskers represent standard deviation.

**Figure 5:**
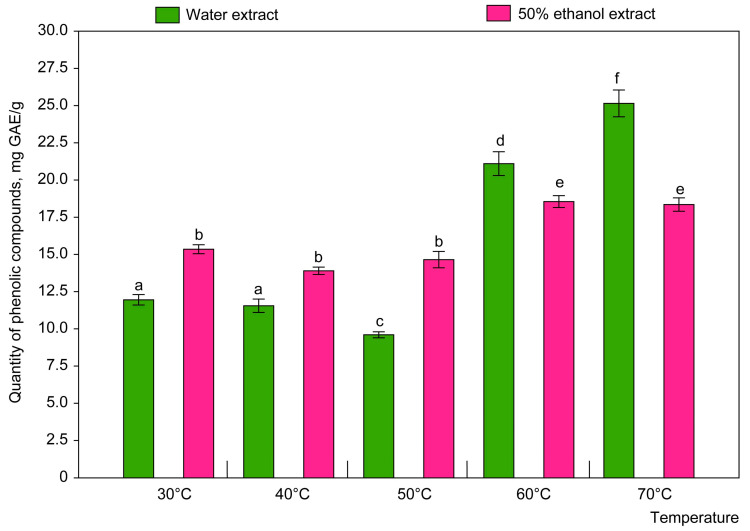
Effect of temperature on quantity of phenolic compounds extracted from lavender herb. Different letters above bars indicate significant differences (*p* < 0.05) between variants. Whiskers represent standard deviation.

Furthermore, the technological parameters were compared to justify the choice of the optimal technology for obtaining extracts with the maximum yield of substances with of rational approach. Three different extractants were used: water, 50% ethanol and 80% ethanol. Different ratios of HRM to extractant (1:10, 1:20 and 1:30) were tested (Figure 4), along with different extraction times in the ultrasonic bath (10, 20, 30 and 60 min) and different extraction temperatures (from 30 °C to 70 °C) (Figure 5). For each extract, the yield of phenolic compounds was determined by the Folin–Ciocalteu assay and recalculated per one gram of HRM for discussion.

The lowest yield of phenolic compounds (Figure 4) was found at an HRM-to-extractant ratio of 1:30. The highest yield of lavender polyphenols was observed using the (1:10) and (1:20) ratios, although the difference in indicators is not statistically significant. In addition, the (1:10) ratio is more rational, not only because of the lower volume of extractant used, but also because of the lower transfer of ballast substances into the extract, which significantly reduces the cost of purification and increases the economic efficiency of the process. Thus, both LWE and LEE (50% ethanol) had the highest polyphenol content, which led to the establishment of optimal extraction conditions specifically for these extracts; namely, for LWE, the optimal conditions were the ratio of HRM to extractant of 1:10, the extraction time of 20 min and the temperature of 70 °C (Figure 5). Overall, the total phenolic content in the herb samples, ranked from the highest to the lowest yield, was as follows: 50% ethanol > water > 80% ethanol.

The results of the two-way permutation analysis of variance showed that the type of solvent (F = 13.67 *df* = 2, *p* = 0.0001) and time of extraction (F = 6.63, *df* = 3, *p* = 0.0004) had a significant effect on the quantity of phenolic compounds extracted, whereas the interaction between the time of extraction and the type of solvent had no significant effect (F = 1.44; *df* = 6, *p* = 0.2127). The ratio of solvent to plant material also had a significant effect (F = 7.46, *df* = 2, *p* = 0.0009), while extraction time (F = 1.98, *df* = 3, *p* = 0.1230) and the interaction of both factors (F = 0.35, *df* = 6, *p* = 0.9035) were insignificant. The analysis also showed that the type of solvent and the ratio of solvent to plant material had a significant effect (F = 287.29, *df* = 2, *p* = 0.0001 and F = 52.45, *df* = 2, *p* = 0.0001, respectively) on the quantity of phenolic compounds in the extract. The effect of the interaction of these two factors on the quantity of phenolic compounds was also significant (F = 14.47, *df* = 4, *p* = 0.0001).

The evaluation of the effect of extraction temperature and extractant on the content of phenolic compounds in the extract by two-way permutation analysis of variance showed that the effect of the extractant was insignificant (F = 2.40, *df* = 1, *p* = 0.141), whereas the effect of the temperature was significant (F = 499.89, *df* = 4, *p* = 0.0001). The interaction between the two factors also had a significant effect on the content of phenolic compounds in the extract (F = 135.20, *df* = 4, *p* = 0.0001).

These results confirm the rationale for further research on lavender herb and support its extensive and rational use in the pharmaceutical industry.

### 3.2. Docking Study Data

The results of the affinity prediction of biologically active substances (BASs) from lavender herb with biotargets are shown in Table 2 and Table 3. For activity modelling, phenolic compounds that were tentatively identified in lavender herb samples [35] were selected for analysis.

**Figure 6:**
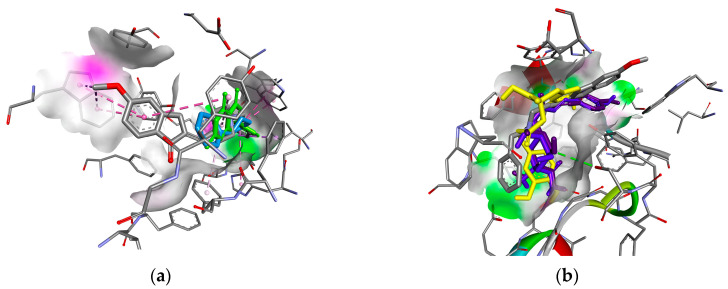
Combined conformational arrangement of the reference inhibitor donepezil (grey) and (**a**) apigenin (green) and 6,7-dihydroxyisoflavone (blue), and (**b**) chlorogenic (purple), neochlorogenic (blue) and rosmarinic acids (yellow) in the active site of AChE.

**Figure 7:**
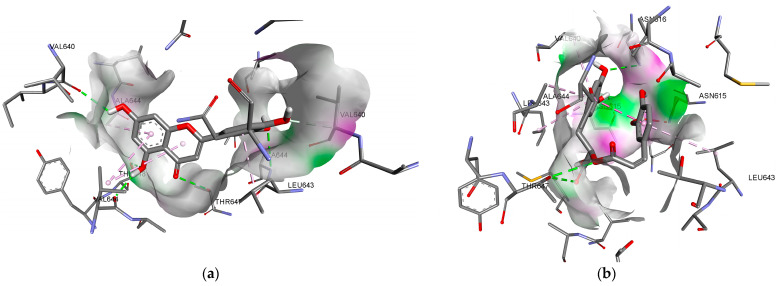
Interaction of luteolin (**a**) and rosmarinic acid (**b**) with amino acid residues of the NMDAR inhibitor site.

The visualisation of the interaction of the studied ligands with the amino acid residues of the active sites of the biotargets and the conformational arrangement relative to the reference ligands are shown in Figure 6 and Figure 7.

### 3.3. HPLC Analysis of Lavender Extracts

**Figure 8:**
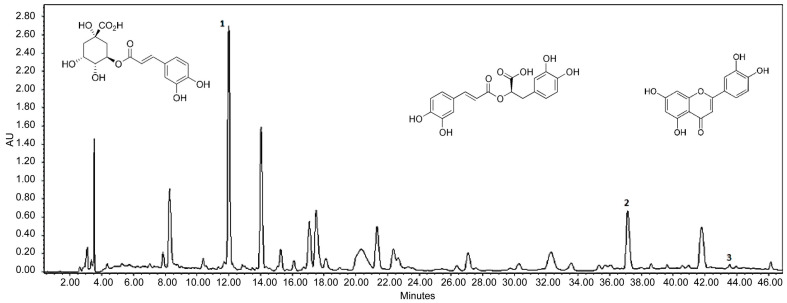
Typical HPLC chromatogram of lavender herb detected at 325 nm: 1—chlorogenic acid (RT 11.64 min); 2—rosmarinic acid (RT 37.18 min); 3—luteolin (RT 43.37 min).

HPLC was used to identify the chemical profile of lavender herb, and the following compounds were identified: chlorogenic acid (retention time: 11.5 min), rosmarinic acid (37 min) and luteolin (43 min) (Figure 8). The compounds identified contribute to the antioxidant effects of lavender herb and consequently to its neuroprotective activity.

The analysis of the content values of the target substances revealed the presence of rosmarinic acid, chlorogenic acid and luteolin in the samples studied. In particular, LEE at an extraction temperature of 60 °C showed one of the highest content values for these acids, with 31.11 mg/g for rosmarinic acid and 1.64 mg/g for chlorogenic acid. Furthermore, the quantity of luteolin was significantly higher in the liquid extracts compared with the dry extracts. The highest concentrations of luteolin were found in LEE (60 °C) and LWE (30 °C), with 0.23 mg/g and 0.21 mg/g, respectively (Table 4).

### 3.4. Antioxidant and Neuroprotective Effects

Antioxidant activity supports neuroprotective activity by combating oxidative stress, a key factor in neuronal damage and degeneration. Therefore, antioxidants are often investigated as potential neuroprotective agents.

**Figure 9:**
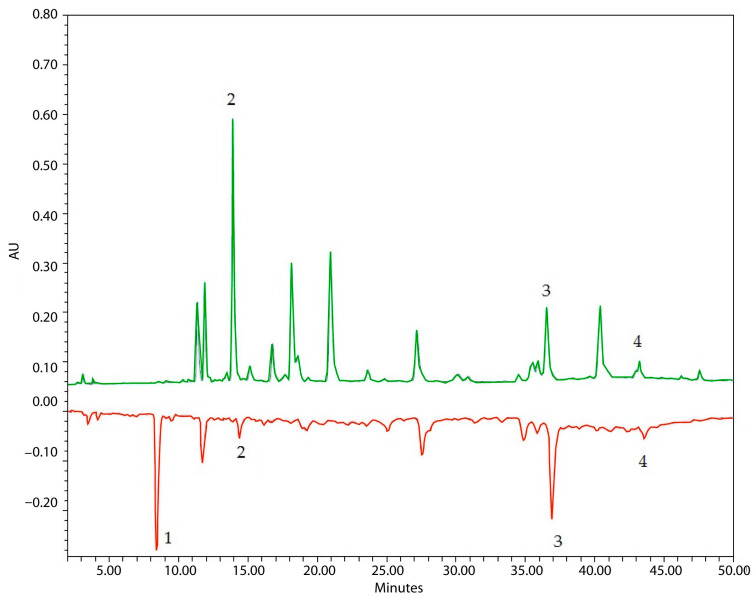
Typical HPLC-ABTS chromatogram of lavender herb extract (LLEE, 50% ethanol) detected at 325 nm (HPLC, green) and 650 nm (ABTS, red): 1—unknown compound; 2—chlorogenic acid; 3—rosmarinic acid; 4—luteolin.

The antioxidant activity of lavender extracts was evaluated by using the HPLC-ABTS method (Figure 9), a robust analytical approach for the identification and quantification of active antioxidant components. This method allowed for the separation of individual compounds and the assessment of their specific contributions to the overall antioxidant potential of the extract. The results highlight the presence of key bioactive constituents, their relative proportions and their ability to neutralise free radicals, providing insights into the potential therapeutic value of lavender in combating oxidative stress. The results of the total antioxidant activity and the activity of individual compounds are presented in Table 5.

A total of six peaks were observed on the ABTS chromatogram, with rosmarinic and chlorogenic acids showing the highest antioxidant activity. In the ethanol extract, luteolin was also identified but showed low activity (1.63 mg/g). Notably, a compound with high antioxidant activity (15–19 mg/g) was detected at 8.4 min, although it was not visible in the spectrum and could not be identified by HPLC analysis. Its high activity suggests that it may be a terpenoid present in low quantities but with significant antioxidant potential. Overall, the activity of both extracts is approximately equivalent, due to the synergistic effects of all components.

The next in vivo experiment is part of a screening to identify the neurotropic effects of lavender derivatives. This experiment was not designed to investigate dose-dependent effects or to evaluate the efficacy of the samples under pathological conditions. Instead, it focuses on selecting the most promising samples for further research in Alzheimer’s disease models. In these models, lavender is administered intraperitoneally at doses ranging from 100 mg/kg to 200 mg/kg [25,26]. As oral administration would reduce bioavailability in the current study, the oral dose was increased to 300 mg/kg to compensate for this. This dose was used as a screening dose for all samples, as the study did not include pathological conditions. Following this screening process, the most promising samples were selected for further investigation and the two leading samples that showed the most significant neuroprotective potential were further investigated.

The results from the Morris Water Maze assay indicated that the lavender extracts studied at a dose of 300 mg/kg did not significantly affect spatial memory in intact animals (Table 6). However, it should be noted that the highest percentage of animals successfully completing the test (83.3%) was observed in the group that received LLEE (50% ethanol).

Similarly, the absence of a significant effect of the extracts on the formation and reproduction of memory in the experimental animals was confirmed by the Conditioned Passive Avoidance Reflex (CPAR) test (Table 7). In addition, no significant effect on cognitive functions was observed in the Extrapolation Release test (Table 8).

The results from the Open Field assay showed that the administration of the test substance LLWE at a dose of 300 mg/kg resulted in a significant reduction in the overall activity of the test animals, affecting both locomotor and exploratory responses, as well as psycho-emotional behaviour. In contrast, the administration of the test substance LLEE (50% ethanol) resulted in a significant decrease only in the number of vertical stands, holes explored and grooming actions (Table 9).

In addition, the inhibitory effects of the extracts on the central nervous system were evident in the Light–Dark Box assay, where both extracts significantly reduced the time spent in the dark chamber while proportionally increasing the time spent in the light chamber (Table 10). However, in the Elevated Plus Maze assay, anxiolytic activity was confirmed only for the LLWE sample (Table 11).

## 4. Discussion

Considering the significant influence of external factors on the cultivation and harvesting of plants [36], as well as the technological processes involved in the extraction of bioactive compounds [37], the current study used a phased approach based on ‘Quality by Design’ principles for accurate planning and goal achievement (Figure 1). Environmental factors strongly influence plant composition, which led to a previous study [35] aimed at identifying optimal regions in Ukraine for growing and harvesting raw materials. Based on chemical composition data [35], the Lviv region was selected as the most promising region for the collection of lavender herb raw material.

This study focused on establishing the extraction technology conditions to increase the yield of bioactive compounds from lavender herb, thereby assessing the pharmacological activity. Lavender is a valuable resource for the pharmaceutical and medical industries. By optimising the extraction process of the bioactive compounds from lavender, it is possible to produce a product rich in active components, ensuring a potent pharmacological effect from the plant.

### 4.1. Extract Preparation and Chemical Analysis

In the technology of phytochemical preparations, several key factors significantly influence the quality of plant extracts and the efficiency of the extraction process. These factors include the type of extractant, the ratio of HRM to extractant, the extraction method, the duration, the temperature, the hydrodynamic conditions and the degree of grinding of the HRM [38,39]. Therefore, we have identified the optimal extraction conditions specifically for lavender to increase the yield of phenolic compounds from HRM.

The selected extractant should selectively extract the desired BAS while being chemically and pharmacologically inert, stable, affordable and cost-effective. It should also inhibit microbial growth and meet safety requirements [37].

This study focuses on the analysis of phenolic compounds in lavender herb, which are inherently hydrophilic and contribute to its neuroprotective activity [40]. Hydrophilic substances are effectively extracted by using solvents with a high dielectric constant; therefore, polar solvents, specifically water and ethanol, were chosen as extractants. The literature indicates that 50% and 80% water–ethanol mixtures are commonly used for the extraction phenolic compounds [38,41,42].

An important step in the extraction process is to determine the time required for the system to reach dynamic equilibrium. Traditional extraction methods often require considerable energy and time, involve the use of expensive and potentially toxic organic solvents and can lead to the transfer of excessive amounts of ballast substances into the extract. This complicates the purification stage of the extraction process [43]. It is, therefore, advisable to consider more efficient technologies, such as ultrasound-assisted extraction (UAE).

Ultrasonic extraction offers several advantages over conventional methods: it significantly improves process efficiency, is considered a ʹgreenʹ technology, allows for a wide range of extractants and is linearly scalable. UAE is a simple and reliable extraction method, as the collapse of cavitation bubbles facilitates particle disruption, resulting in the release of a greater quantity of BASs [44,45,46,47]. The literature indicates that the extraction time for phenolic compounds using ultrasonic waves typically ranges from 10 to 60 min [48,49,50,51,52].

Temperature control is crucial to reducing the extraction time and increase the yield of BASs from the HRM. The choice of the temperature regime depends primarily on the boiling point of the extractant and the thermal stability of the extracted BASs. Phenolic compounds are generally considered to be thermostable, allowing for their extraction over a wide temperature range [53,54].

To determine the optimal extraction conditions, lavender extracts obtained from different types of HRM and by using different extraction conditions, namely, extractant, ratio of HRM to extractant, extraction temperature and time, were analysed. The parameter used to determine the extraction efficiency was the yield of the sum of phenolic compounds according to the Folin–Ciocalteu method. The results obtained are shown in Figure 3, Figure 4 and Figure 5.

The highest yield of phenolic compounds from lavender herb was obtained by using purified water and 50% ethanol as extractants (Figure 4), due to their high dielectric constants (78.3 mg/g for water and 49.1 mg/g for 50% ethanol). According to the analysis results (Figure 5), the highest yield of phenolic compounds was observed with the 1:20 ratio of HRM to extractant. However, for the extraction of polyphenols from lavender herb, both the yield of BASs and the preservation of high concentrations of active compounds in the extracts are of great importance. Thus, the use of a ratio of 1:10 makes it possible to obtain more concentrated extracts with no less high yield of BASs than extracts obtained with a ratio of 1:20, due to the use of half the quantity of extractant. This significantly reduces the cost of the extractant, making the process more efficient and economically viable. It should also be noted that the quantity of ballast passing into the water and ethanolic extracts will be greater with the 1:20 ratio, as a greater volume of extractant provides greater solubility. Ballast materials can in turn affect the stability and quality of the final extract, requiring additional effort to remove them. The optimum extraction conditions were, therefore, found to be a ratio of HRM to extractant of 1:10, an extraction time of 20 min and temperatures of 70 °C and 60 °C for water and ethanolic extracts, respectively. As the use of 80% ethanol resulted in the lowest yield of bioactive compounds, this extractant was excluded from further studies. This highlights the importance of selecting appropriate extraction conditions to increase the efficiency of the extraction of phenolic compounds from lavender herb.

It has also been experimentally proven that the separation of raw materials of small herbs into leaves, stems and inflorescences is not recommended, since water and ethanolic extracts of lavender stems contained two times less compounds (Figure 3) than flowers or whole herb. Therefore, considering the need to optimise the technological process, it is not reasonable to separate the raw material.

These results agree with the study of Blažeković and coauthors [55], who reported that the content of phenolic compounds in lavender raw materials decreased in the following order: leaves > flowers > inflorescence stems. Therefore, it is expected that the herb, which includes all parts of the plant, would have a higher total content of phenolic compounds compared with the individual parts (Figure 3).

As the rate of transition of BASs into the extract is also influenced by temperature, the next phase of our research focused on determining the optimal extraction temperature. It is known that higher extraction temperatures can enhance the interaction between the solvent and the HRM, as well as improve diffusion processes and solubility [42,56]. In this study, we evaluated a temperature range from 30 °C to 70 °C while keeping other experimental parameters constant (Figure 5). In the temperature range 30–50 °C, the ethanolic extractant showed superior extraction capacity, extracting 1.2–1.5 times more BASs from the HRM compared with LWE. Interestingly, within this temperature range, the content of phenolic compounds decreased with the increase in temperature for both extracts, with the order being 30 °C > 40 °C < 50 °C. Conversely, LWE showed the highest concentration of BASs at higher temperatures. This suggests that an increase in extraction temperature facilitates the rupture of plant cell walls, thereby increasing the diffusion of phenolic compounds into the water. Thus, the most optimal extraction temperature for LWE was found to be 70 °C, as this temperature gave the highest yield of phenolic compounds (Figure 5). Similarly, the best release of BASs in LEE was observed at elevated temperatures of 60 °C and 70 °C. Since the quantities extracted at these two temperatures were not significantly different, 60 °C was chosen as a rational extraction temperature for 50% ethanol extraction.

It is well known that the effects of plant constituents on the central nervous system are typically determined by the total sum of BASs [57,58]. However, the ratio of individual compounds or the presence of specific pharmacological markers can significantly influence biological activity.

Therefore, for the subsequent justification of the technology, we decided to pay attention not only to the total yield of phenolic compounds but also to their profile. Therefore, we tried to find out which of the phenolic compounds previously found in lavender herb [3,59,60] exhibited affinity with the active sites of biotargets involved in neuroprotective activity by molecular docking. These compounds will serve as markers for the further selection of extraction conditions and subsequently for the standardisation of extracts.

For this prediction, we focused on two key neuroregulatory vectors that can influence cognitive function and help prevent neurodegeneration: cholinergic and glutamatergic systems.

According to the results obtained (Table 2 and Table 3), among the phenolic compounds of lavender herb, the highest predicted affinity with the acetylcholinesterase (AChE) inhibitor site was observed for apigenin and its glycoside, luteolin; ononin (binding energy < −10.0 kcal/mol); chlorogenic, neochlorogenic and rosmarinic acids; hyperoside; and 6,7-dihydroxyisoflavone (binding energy < −9.2 kcal/mol). However, it is noteworthy that the scoring function values of all these compounds were somewhat lower than those of the reference ligands, donepezil (−11.0 kcal/mol) and BPMI (−11.3 kcal/mol).

Detailed analysis of the interactions with peptide residues and the conformational arrangements relative to donepezil revealed that apigenin, 6,7-dihydroxyisoflavone and luteolin were unable to fully occupy the active-site cavity. The glycoside of apigenin was stabilised in the cavity solely by hydrogen bonding (Figure 6a). In contrast, a successful arrangement was predicted for chlorogenic, neochlorogenic and rosmarinic acids, which entered into hydrophobic interactions with the phenylalanine (Phe330, 331) and tryptophan (Trp84, 279) residues that are involved in the binding of the reference ligand (Figure 6b). This conformational arrangement suggests a high probability of exhibiting inhibitory activity against acetylcholinesterase.

Among lavender phenolic compounds, the degree of affinity with the NMDA receptor inhibitor site was found to be higher for rosmarinic, neochlorogenic, and chlorogenic acids, as well as for luteolin, 6,7-dihydroxyisoflavone and apigenin, compared with the reference ligand memantine (binding energy < −5.8 kcal/mol). Key hydrophobic interactions with alanine (Ala644), leucine (Leu643) and valine (Val644) residues play a crucial role in binding within the channel pore, facilitating the manifestation of inhibitory effects of the ligand [23]. For luteolin, a strong fixation of all molecular fragments was predicted, facilitated by hydrophobic bonds with the amino acid residues (Figure 7a). In the case of 6,7-dihydroxyisoflavone, only a hydrophobic interaction with leucine (Leu643) was predicted, whereas apigenin showed interactions with leucine (Leu643) and alanine (Ala644) through its pyran fragment, undermining the possibility of achieving a stable conformation.

The results of the molecular docking of the BACs tentatively identified in lavender herb indicate that chlorogenic acid and rosmarinic acid may have the highest probability of inhibiting the enzyme acetylcholinesterase (AChE). The NMDA receptor antagonism is lower for lavender phenolic compounds, of which rosmarinic acid and luteolin are the most potent. Therefore, chlorogenic acid, rosmarinic acid and luteolin were selected as possible specific activity markers for further studies.

A targeted analysis focusing on the extraction of individual BASs was carried out by using HPLC with the aim of determining the optimum extraction temperature for LWE and LEE. The analysis focused on hydroxycinnamic acids, specifically chlorogenic and rosmarinic acids, and the flavonoid luteolin. The contents of these markers were evaluated at a minimum temperature of 30 °C, together with 60 °C for ethanolic extracts and 70 °C for water extracts, identified in previous studies as optimal for the extraction of phenolic compounds.

The results (Table 4) showed significant differences in the quantities of rosmarinic and chlorogenic acids extracted, depending on the type of extractant and its temperature. Rosmarinic acid was not detectable in LWE obtained at 30 °C. However, raising the temperature to 70 °C significantly improved the extraction process in water, resulting in the extraction of this acid at 7.08 mg/g. The use of 50% ethanol as the extractant resulted in a significant increase in rosmarinic acid content, 3.6 times higher at 30 °C and 4.4 times higher at 60 °C. The yield of chlorogenic acid also increased with the increase in temperature for both extractants. Interestingly, the luteolin content was higher at the lower temperature (30 °C) in LWE, whereas it was higher at the higher temperature (60 °C) in LEE.

The choice of temperatures of 60 °C for the 50% ethanolic extract (LEE) and of 70 °C for the water extract (LWE) was validated as it ensures a high degree of extraction of BASs from lavender. The results are in line with the existing literature, which indicates that the highest yields of phenolic compounds are typically observed at elevated temperatures, around 60–80 °C [53,54,61,62].

From a technological point of view, it is advisable to use dry extracts to produce solid dosage forms containing plant constituents. It was, therefore, decided to convert the liquid extracts into dry extracts by lyophilisation. The resulting dry extracts were evaluated for several parameters, including their description, moisture content and phenolic compound content.

The lyophilised dry extracts obtained were found to be free-flowing, homogeneous and hygroscopic powders, each with a characteristic plant aroma. There was a slight variation in colour, ranging from light brown to dark brown. Both extracts complied with the moisture content specifications of the European Pharmacopoeia, which requires a moisture content of less than 5% [63]. The extraction yields were 13% ± 0.44% for the LLWE and 14% ± 0.24% for the LLEE (50% ethanol). Differences in component composition were observed between lyophilised and non-lyophilised extracts, probably related to the effect of the lyophilisation process on the composition and stability of the extracts. Lyophilisation or freeze-drying is known to preserve bioactive compounds while minimising thermal degradation and oxidation. However, it can also lead to changes in the concentration of certain compounds due to the sublimation of water and other volatiles. In the context of our results, differences in marker levels (for rosmarinic acid content, 2.8 mg/g lower in LLWE than in LWE, Table 4) may reflect changes in compound stability, solubility or interactions during the lyophilisation process. These effects are particularly relevant for phenolic compounds and terpenoids, which are sensitive to changes in processing conditions.

Despite the slightly lower content of marker substances in the lyophilised extract compared with the non-lyophilised one, the use of lyophilisation to obtain dry extracts remains justified because of the preservation of the bioactivity of all components, the stability of the substances obtained and the possibility of standardising substances due to the constancy of the composition. These advantages make lyophilisation the preferred method for obtaining dry extracts, especially when the stability and bioactivity of the compounds are priorities.

### 4.2. Antioxidant and Neuroprotective Effects of Lavender Extracts

Research into the neuroprotective potential of lavender is highly relevant in the current context of Ukraine, where the population is exposed to high levels of constant stress. Increased anxiety, nervousness, sleep disturbances, nightmares, depression and the prevalence of neurodegenerative diseases such as Parkinson’s and Alzheimer’s are becoming more common [64,65,66]. Such conditions require effective therapeutic interventions, and lavender as an herbal medicine represents a promising option.

Antioxidant capacity is often used as a marker when evaluating the potential of plants or natural products, such as lavender, for neuroprotective properties. Measuring antioxidant levels can indicate whether a substance can reduce oxidative stress, which is strongly associated with cognitive decline and neurodegenerative disorders.

In our study, the higher neuroprotective activity of the water extract, despite its lower phenolic content, confirms the role of synergy between its components. These results suggest that although phenolic compounds alone may not be the primary therapeutic agents, their combined action with other bioactive compounds may enhance neuroprotective outcomes. This is consistent with the broader view that natural compounds, including phenolic compounds, may serve as complementary or adjuvant therapies rather than as direct replacements for alkaloid-based drugs.

Historically used in herbal medicine to calm the nervous system, lavender is now being researched for its neuroprotective potential. Its antioxidant properties are one of the key mechanisms by which it may support cognitive health, help prevent neurodegeneration and improve overall brain function.

Previous studies have linked improved neurological function and reduced blood–brain barrier permeability in rats with enhanced endogenous antioxidant defences and the inhibition of oxidative stress in the brain [11]. Research has also confirmed the efficacy of lavender in treating memory problems, anxiety and depressive behaviour [12,14], and in promoting structural and functional recovery following traumatic brain injury [13]. A significant advantage of lavender as a remedy is its botanical origin, which tends to have fewer side effects than synthetic drugs. Lavender Lyophilised Water Extract (LLWE) showed remarkable anxiolytic and sedative properties, as evidenced by a significant reduction in the sum of exploratory responses, emotional responses and total activity in the Open Field test. In addition, there was a significant increase in the time spent in the unlit area in the Elevated Plus Maze test. In contrast, the activity of the lyophilised ethanol extract of lavender was less pronounced. Of the behavioural responses measured in the Open Field test, only vertical standing, explored holes and grooming showed significant decreases. In the Light–Dark Box test, there was a significant reduction in the time spent in the unlit areas, indicating the differential efficacy of the two extracts.

None of the extracts affected memory function in the Morris Water Maze and Conditioned Passive Avoidance Reflex assays, nor did they affect cognitive properties in the Extrapolation Release test. This suggests both the absence of relevant activities in the samples and the absence of significant CNS depressant or amnesiogenic effects, which serves as a safety factor for herbal anxiolytics. Furthermore, the results of our tests showed higher efficacy of the water extract compared with the ethanolic extract, which correlates with previous research on water lavender extract for the treatment of stress-induced depression [14]. Thus, the pharmacological tests are consistent with the results of other studies, confirming a sufficient level of neuroprotective activity in lavender extracts, particularly the water extract, and suggesting their potential efficacy in the treatment of nervous system disorders. The high levels of rosmarinic acid and chlorogenic acid contribute to its neuroprotective effects. It is very important to create and maintain optimal growth conditions for medicinal plants [67], as this makes it possible to maintain a constant composition of active substances in the raw material, optimise the production of extracts and increase their therapeutic efficacy.

## 5. Conclusions

The present study highlights the promising antioxidant and neuroprotective properties of the polyphenol-rich extracts of lavender cultivated in Ukraine. Due to the presence of rosmarinic acid and chlorogenic acid, the extracts showed significant antioxidant activity, which is crucial to mitigate oxidative stress, a key factor in neurodegenerative diseases such as Alzheimer’s and Parkinson’s diseases. In addition, the neuroprotective effects observed suggest the potential therapeutic utility of these extracts in preserving cognitive function and reducing neuronal damage. Certainly, further studies on biochemical parameters associated with inflammation and/or neuroprotection, including in vitro and in vivo studies in additional models, are needed to confirm the primary results obtained and to explore the full therapeutic potential of lavender by-products in neurological disorders. This will promote the widespread use of lavender herb in the future through the development of innovative pharmacologically active compounds.

## Figures and Tables

**Table 1:** The main validation characteristics of reference phenolic compounds.

Component	RT (min)	Coefficient of Determination *R*^2^	Equation, y	Linearity Range (µg/mL)	LoD (µg/mL)	LoQ (µg/mL)	Repeatability RT/Area (%)	Precision RT/Area (%)
Chlorogenic acid	11.95	0.99980	3.10 × 10^4^x − 7.62 × 10^3^	0.406–208.200	0.049	0.147	0.2/0.6	0.4/0.8
Rosmarinic acid	37.16	0.99985	6.77 × 10^3^x − 1.87 × 10^3^	1.953–500.0	0.061	0.487	0.2/0.5	0.3/0.9
Luteolin	43.38	0.99990	4.23 × 10^4^x − 3.89 × 10^2^	0.214–110.0	0.053	0.195	0.1/0.4	0.15/0.7

**Table 2:** Results of prediction of affinity of biologically active compounds isolated from lavender herb and native ligands with active sites of biotargets.

Ligand	Biotarget	
	AChE (5NAU)	NMDA (7SAD)
BPMI	−11.3 kcal/mol	–
Donepezil	−11.0	–
Memontin	–	−5.8
Safinamid	–	–
Apigenin	−10.8	−7.8
Caffeic acid	−7.2	−6.4
Chlorogenic acid	−9.2	−7.4
Coumaric acid	−7.2	−5.3
Ferulic acid	−7.6	−5.5
Gallic acid	−6.4	−5.0
Hyperoside	−9.4	−5.5
6,7-Dihydroxyisoflavone	−9.2	−7.2
Luteolin	−10.3	−7.2
Neochlorogenic acid	−9.2	−7.4
Ononin	−10.6	−0.4
Rosmarinic acid	−9.7	−7.3
Vanillic acid	−6.2	−5.0
Vanillin	−6.1	−4.8

**Table 3:** Types of interaction of the studied ligands with the best values of scoring functions with the amino acid residues of the biotargets. Letters in superscript: (^a^) hydrogen bond; (^b^) hydrophobic bond.

Biotarget	Compound	Interaction with Amino Acids
AChE (5NAU)	Ononin	^a^: Phe330 and Trp279; ^b^: Tyr121(3), Tyr130, Glu199, Trp84, Gly118, Ser200 and Gly335.
	Rosmarinic acid	^a^: Trp84, Phe330 and Phe331; ^b^: His440, Ser81, Glu199, Tyr130 and Gly441.
	Chlorogenic acid	^a^: Phe331 and Trp279; ^b^: Ser122, Ser200, Phe288 and Phe331.
NMDA (7SAD)	Luteolin	^a^: Ala644, Val644 and Leu643; ^b^: Ala644, Thr647 and Val640(2).
	Rosmarinic acid	^a^: Val640, Leu643, Ala644 and Leu643, ^b^: Asn616, Thr647(2) and Asn615.

**Table 4:** Quantitative contents of marker compounds in lavender herb extracts by HPLC method.

Extract Type	Quantity, mg/g		
	Rosmarinic Acid	Chlorogenic Acid	Luteolin
LLWE 1:10 (70 °C) 20 min	3.34 ± 0.06	1.17 ± 0.02	0.14 ± 0.002
LLEE (50% ethanol) 1:10 (60 °C) 20 min	28.31 ± 0.50	1.30 ± 0.02	0.20 ± 0.004
LWE 1:10 (70 °C) 20 min	7.08 ± 0.12	1.46 ± 0.03	0.08 ± 0.001
LWE 1:10 (30 °C) 20 min	not detected	1.23 ± 0.02	0.21 ± 0.004
LEE (50% ethanol) 1:10 (60 °C) 20 min	31.11 ± 0.55	1.64 ± 0.03	0.23 ± 0.004
LEE (50% ethanol) 1:10 (30 °C) 20 min	25.37 ± 0.45	1.45 ± 0.03	0.20 ± 0.004

**Table 5:** Radical scavenging activity of individual compounds of lavender extracts expressed as TEAC (mg/g) using ABTS post-column assay.

Peak No.	Component	RTime	LLWE	LLEE
1	Unknown compound	8.402	19.26 ± 0.34	15.57 ± 0.28
2	Chlorogenic acid	11.737	4.87 ± 0.09	4.91 ± 0.09
3	Rosmarinic acid	36.935	9.37 ± 0.18	13.42 ± 0.24
4	Luteolin	43.570	-	1.63 ± 0.03
Total			55.90 ± 0.96	50.85 ± 0.98

**Table 6:** Effects of test sample on spatial memory of rats in Morris Water Maze test (median [Q25; Q75], *n* = 6).

Experimental Group	Latent Time of Platform Location, s	Time Spent in Quadrant with Platform, %	Number of Animals That Passed Decision Test, %
Negative control	97.5 [68; 120]	28.86 [22.33; 34.17]	66.7
LLWE	101.5 [76; 120]	25.83 [18.46; 31.33]	50.0
LLEE	93.5 [76; 112]	26.19 [22.62; 30.00]	83.3

Note: s—seconds.

**Table 7:** Effects of test sample on formation of Conditioned Passive Avoidance Reflex in intact rats (median [Q25; Q75], *n* = 6).

Experimental Group	Latency of Chamber Entry in Training Phase, s	Latency of Chamber Entry in Test Phase, s	Animals with Formed CPAR, %
Negative control	23.5 [19; 28]	138.5 [96; 180]	33.3
LLWE	23.0 [20; 29]	156.0 [82; 180]	33.3
LLEE (50% ethanol)	20.5 [14; 28]	134.0 [73; 180]	33.3

Note: s—seconds.

**Table 8:** Effects of test sample on performance of intact rats in Extrapolation Release test (median [Q25; Q75], *n* = 6).

Experimental Group	Time to Complete Task, s	Animals That Completed Task, %
Negative control	77.0 [54; 102]	83.3
LLWE	57.5 [41; 140]	83.3
LLEE (50% ethanol)	40.5 [32; 166]	83.3

Note: s—seconds.

**Table 9:** Effects of test sample on behavioural responses of rats in Open Field test (median [Q25; Q75], *n* = 6).

Indicator Under Study	Experimental Group		
	Negative Control	LLWE	LLEE
Open Field test			
Squares crossed	40.0 [28; 44]	18.5 [13; 37] *	39.0 [11; 42]
Vertical stands	9.0 [5; 11]	6 [0; 7]	1.0 [0; 4] *
Holes explored	7.0 [5; 9]	1 [0; 1] *	0.0 [0; 1] *
Sum of exploratory responses	53.5 [46; 61]	25 [17; 46] *	43 [11; 46]
Defecations	1.0 [1; 1]	0.0 [0; 0]	1.0 [0; 1]
Urinations	1.0 [0; 1]	0.0 [0; 0]	1.0 [0; 1]
Grooming acts	2.5 [1; 3]	0.0 [0; 1] *	0.5 [0; 1] *
Sum of emotional responses	4.5 [3; 5]	1.0 [0; 1] *	2.0 [1; 3]
Sum of all activities	57 [50; 66]	26.5 [18; 47] *	45.5 [11; 48]

Note: *—statistically significant differences compared with the negative control group, *p* < 0.05.

**Table 10:** Effects of test sample on behavioural responses of rats in Light–Dark Box test (median [Q25; Q75], *n* = 6).

Experimental Group	Number of Transitions Between Chambers	Time Spent in Light Chamber, s	Time Spent in Dark Chamber, s
Negative control	2.5 [1; 4]	70.0 [52; 84]	230.0 [216; 248]
LLWE	2.5 [2; 3]	147.0 [95; 203] *	153.0 [97; 205] *
LLEE (50% ethanol)	2.5 [1; 4]	102.0 [68; 157] *	198.0 [143; 232] *

Note: *—statistically significant differences compared with the negative control group, *p* < 0.05.

**Table 11:** Effects of test sample on behavioural responses of rats in Elevated Plus Maze test (median [Q25; Q75], *n* = 6).

Indicator Under Study	Experimental Group		
	Negative Control	LLWE	LLEE
Elevated Plus Maze test			
Dark chamber entry latency, s	29.5 [27; 33]	39.0 [34; 42]	38.5 [26; 82]
Time spent in centre of maze, s	25.5 [19; 27]	21.0 [18; 25]	26.0 [15; 28]
Time spent in closed arm, s	212.0 [194; 232]	164.5 [137; 188] *	178.5 [77; 225]
Time spent in open arm, s	59.5 [41; 71]	119.0 [90; 138] *	94.0 [63; 195]
Number of transitions between arms	7.5 [6; 9]	8.0 [7; 10]	8.5 [7; 9]

Note: *—statistically significant differences compared with the negative control group, *p* < 0.05.

## Data Availability

All data are included in the article.

## Abbreviations

ABTS	2,2′-azino-bis(3-ethylbenzothiazoline-6-sulfonic acid
BAS	biologically active substance
BPMI	(2E)-2-[(1-benzyl-4-piperidyl)methylene]-5-methoxy-indan-1-one
CNS	central nervous system
CPAR	Conditioned Passive Avoidance Reflex
GA	gallic acid
HPLC	High-Performance Liquid Chromatography
HRM	herbal raw material
i.p.	intraperitoneal
LEE	Lavender Ethanolic Extract
LLEE	Lavender Lyophilised Ethanolic Extract
LLWE	Lavender Lyophilised Water Extract
LWE	Lavender Water Extract
QbD	Quality by Design
RMSD	root-mean-square deviation
SCI	spinal cord injury
UAE	ultrasonic-assisted extraction

## References

[1] Niedzielska E., Smaga I., Gawlik M., Moniczewski A., Stankowicz P., Pera J., Filip M. (2016). Oxidative stress in neurodegenerative diseases. Mol. Neurobiol..

[2] Batiha G.E., Teibo J.O., Wasef L., Shaheen H.M., Akomolafe A.P., Teibo T.K.A., Al-Kuraishy H.M., Al-Garbeeb A.I., Alexiou A., Papadakis M. (2023). A review of the bioactive components and pharmacological properties of *Lavandula* species. Naunyn Schmiedebergs Arch Pharmacol..

[3] Dobros N., Zawada K.D., Paradowska K. (2022). Phytochemical profiling, antioxidant and anti-inflammatory activity of plants belonging to the *Lavandula* genus. Molecules.

[4] Cavanagh H.M.A., Wilkinson J.M. (2005). Lavender essential oil: A review. Healthc. Infect..

[5] Hui L., He L., Huan L., Xiaolan L., Aiguo Z. (2010). Chemical composition of lavender essential oil and its antioxidant activity and inhibition against rhinitis- related bacteria. Afr. J. Microbiol. Res..

[6] Theophrastus “On Odours”. https://www.loebclassics.com/view/theophrastus-odours/1916/pb_LCL079.327.xml.

[7] Prusinowska R., Śmigielski K. (2014). Composition, biological properties and therapeutic effects of lavender (*Lavandula angustifolia* L). A review. Herba Pol..

[8] Olufunmilayo E.O., Gerke-Duncan M.B., Holsinger R.M.D. (2023). Oxidative stress and antioxidants in neurodegenerative disorders. Antioxidants.

[9] Kotvitska A., Prokopenko O. (2017). The study of Parkinson’s disease main etiological factors. Sci. Pharm. Sci..

[10] Tăbărașu A.M., Anghelache D.N., Găgeanu I., Biriș S.Ș., Vlăduț N.V. (2023). Considerations on the use of active compounds obtained from lavender. Sustainability.

[11] Rabiei Z., Rafieian-Kopaei M. (2014). Neuroprotective effect of pretreatment with *Lavandula officinalis* ethanolic extract on blood-brain barrier permeability in a rat stroke model. Asian Pac. J. Trop. Med..

[12] Rahmati B., Kiasalari Z., Roghani M., Khalili M., Ansari F. (2017). Antidepressant and anxiolytic activity of *Lavandula officinalis* aerial parts hydroalcoholic extract in scopolamine-treated rats. Pharm. Biol..

[13] Kaka G., Yaghoobi K., Davoodi S., Hosseini S.R., Sadraie S.H., Mansouri K. (2016). Assessment of the neuroprotective effects of *Lavandula angustifolia* extract on the contusive model of spinal cord injury in Wistar rats. Front. Neurosci..

[14] Behrad A.G.M., Amir M., Asma N., Zahra K. (2023). Effects of Lavender Aqueous Extract on the Levels of Oxidative Stress Markers in the Sera and Tissues from Male Sprague–Dawley Rats with Chronic Mild Stress Induced Depression. JABS.

[15] (2017). ICH Guideline Q8 (R2): On Pharmaceutical Development, Step 5.

[16] Pérez M., Dominguez-López I., Lamuela-Raventós R.M. (2023). The chemistry behind the Folin-Ciocalteu method for the estimation of (poly)phenol content in food: Total phenolic intake in a Mediterranean dietary pattern. J. Agric. Food. Chem..

[17] Ivanauskas L., Uminska K., Gudžinskas Z., Heinrich M., Georgiyants V., Kozurak A., Mykhailenko O. (2023). Phenological variations in the content of polyphenols and triterpenoids in *Epilobium angustifolium* herb originating from Ukraine. Plants.

[18] (2005). ICH Guidance Q2 (R1): Validation of Analytical Procedures: Text and Methodology. ICH Harmonised Tripartite Guideline.

[19] Marksa M., Radušienė J., Jakštas V., Ivanauskas L., Marksienė R. (2016). Development of an HPLC post-column antioxidant assay for *Solidago canadensis* radical scavengers. Nat. Prod. Res..

[20] Forli S., Huey R., Pique M.E., Sanner M.F., Goodsell D.S., Olson A.J. (2016). Computational protein-ligand docking and virtual drug screening with the AutoDock suite. Nat. Protoc..

[21] RCSB Protein Data Bank (RCSB PDB). https://www.rcsb.org/.

[22] Caliandro A. (2017). Digital methods for ethnography: Analytical concepts for ethnographers exploring social media environments. J. Contemp. Ethnogr..

[23] Chou T.H., Kang H., Simorowski N., Traynelis S.F., Furukawa H. (2022). Structural insights into assembly and function of GluN1-2C, GluN1-2A-2C, and GluN1-2D NMDARs. Mol. Cell..

[24] Directive 2010/63/EU of the European Parliament and of the Council of 22 September 2010. pp. 33–79. https://eur-lex.europa.eu/eli/dir/2010/63/oj.

[25] Arasteh A., Karimpour M., Fallah F., Kiani S., Kakavan M. (2023). Activity of Citrus aurantium and *Lavandula angustifolia* in Alzheimer’s Disease Symptoms in Male Wistar Rats. Avicenna J. Med. Biotechnol..

[26] Zali H., Zamanian-Azodi M., Rezaei Tavirani M., Akbar-Zadeh Baghban A. (2015). Protein Drug Targets of *Lavandula angustifolia* on treatment of Rat Alzheimer’s Disease. Iran J. Pharm. Res..

[27] Semenets A.P., Suleiman M.M., Fedosov A.I., Shtrygol S.Y., Havrylov I.O., Mishchenko M.V., Kovalenko S.M., Georgiyants V.A., Perekhoda L.O. (2022). Synthesis, docking, and biological evaluation of novel 1-benzyl-4-(4-(R)-5-sulfonylidene-4,5-dihydro-1H-1,2,4-triazol-3-yl)pyrrolidin-2-ones as potential nootropic agents. Eur. J. Med. Chem..

[28] Bromley-Brits K., Deng Y., Song W. (2011). Morris water maze test for learning and memory deficits in Alzheimer’s disease model mice. J. Vis. Exp..

[29] Seibenhener M.L., Wooten M.C. (2015). Use of the Open Field Maze to measure locomotor and anxiety-like behavior in mice. J. Vis. Exp..

[30] Walf A.A., Frye C.A. (2007). The use of the elevated plus maze as an assay of anxiety-related behavior in rodents. Nat. Protoc..

[31] Eagle A.L., Wang H., Robison A.J. (2016). Sensitive assessment of hippocampal learning using temporally dissociated passive avoidance task. Bio. Protoc..

[32] Takao K., Miyakawa T. (2006). Light/dark transition test for mice. J. Vis. Exp..

[33] Havrylov I., Shtrygol S. (2021). Investigation of the effect of a modified fragment of neuropeptide Y on memory phases and extrapolation escape of animals. Ceska Slov. Farm..

[34] MS Excel 2007 Basic Package an IBM SPSS Statistics 22. https://www.pubcompare.ai/product/IBoZm48BUt4m_cx6zdiQ/.

[35] Mykhailenko O., Hurina V., Ivanauskas L., Marksa M., Skybitska M., Kovalenko O., Lytkin D., Vladymyrova I., Georgiyants V. (2024). *Lavandula angustifolia* herb from Ukraine: Comparative chemical profile and in vitro antioxidant activity. Chem. Biodivers..

[36] Hassiotis C.N., Ntana F., Lazari D.M., Poulios S., Vlachonasios K.E. (2014). Environmental and developmental factors affect essential oil production and quality of *Lavandula angustifolia* during flowering period. Ind. Crops. Prod..

[37] Zhang Q.W., Lin L.G., Ye W.C. (2018). Techniques for extraction and isolation of natural products: A comprehensive review. Chin. Med..

[38] Mieriņa I., Jakaite L., Kristone S., Adere L., Jure M. (2018). Extracts of peppermint, chamomile and lavender as antioxidants. Key Eng. Mater..

[39] Guo X., Wang P. (2020). Aroma characteristics of lavender extract and essential oil from *Lavandula angustifolia* Mill. Molecules.

[40] Vesna N., Žika L., Mihailo R., Vladic J., Nikolovski B., Adamović D. (2014). Investigation of cultivated lavender (*Lavandula officinalis* L.) extraction and its extracts. Chem. Ind. Chem. Eng. Q.

[41] Zakaria S.M., Kamal S.M.M. (2015). Subcritical water extraction of bioactive compounds from plants and algae: Applications in pharmaceutical and food ingredients. Food Eng. Rev..

[42] Filly A., Fabiano-Tixier A.S., Louis C., Fernandez X., Chemat F. (2016). Water as a green solvent combined with different techniques for extraction of essential oil from lavender flowers. Comptes Rendus Chim..

[43] Nayak A., Bhushan B. (2019). An overview of the recent trends on the waste valorization techniques for food wastes. J. Environ. Manag..

[44] Chemat F., Rombaut N., Sicaire A.G., Meullemiestre A., Fabiano-Tixier A.S., Abert-Vian M. (2017). Ultrasound assisted extraction of food and natural products. Mechanisms, techniques, combinations, protocols and applications: A review. Ultrason. Sonochem..

[45] Olmo-García L., Bajoub A., Benlamaalam S., Hurtado-Fernández E., Bagur-González M.G., Chigr M., Mbarki M., Fernández-Gutiérrez A., Carrasco-Pancorbo A. (2018). Establishing the phenolic composition of *Olea europaea* L. leaves from cultivars grown in Morocco as a crucial step towards their subsequent exploitation. Molecules.

[46] Turrini F., Boggia R., Leardi R., Borriello M., Zunin P. (2018). Optimization of the ultrasonic-assisted extraction of phenolic compounds from *Oryza sativa* L. ‘violet Nori’ and determination of the antioxidant properties of its caryopses and leaves. Molecules.

[47] Aware C.B., Patil R.R., Vyavahare G.D., Gurme S.T., Jadhav J.P. (2019). Ultrasound-Assisted aqueous extraction of phenolic, flavonoid compounds and antioxidant activity of Mucuna macrocarpa beans: Response surface methodology optimization. J. Am. Coll. Nutr..

[48] Tao Y., Zhang Z., Sun D.W. (2014). Kinetic modeling of ultrasound-assisted extraction of phenolic compounds from grape marc: Influence of acoustic energy density and temperature. Ultrason. Sonochem..

[49] Giacometti J., Žauhar G., Žuvić M. (2018). Optimization of ultrasonic-assisted extraction of major phenolic compounds from olive leaves (*Olea europaea* L.) using response surface methodology. Foods.

[50] Mazza K., Santiago M., Nascimento L., Godoy R., Souza E., Brígida A., Borguini R., Tonon R. (2018). Syrah grape skin valorisation using ultrasound-assisted extraction: Phenolic compounds recovery, antioxidant capacity and phenolic profile. Int. J. Food Sci. Technol..

[51] Ismail B.B., Guo M., Pu Y., Wang W., Ye X., Liu D. (2019). Valorisation of baobab (*Adansonia digitata*) seeds by ultrasound assisted extraction of polyphenolics. Optim. Comp. Conv. Methods. Ultrason. Sonochem..

[52] Zardo I., Sobczyk A., Marczak L., Sarkis J. (2019). Optimization of ultrasound assisted extraction of phenolic compounds from sunflower seed cake using response surface methodology. Waste Biomass Valori..

[53] Gironi F., Piemonte V. (2011). Temperature and solvent effects on polyphenol extraction process from chestnut tree wood. Chem. Eng. Res. Des..

[54] Dent M., Verica D.U., Brncic M., Bosiljkov T., Levaj B. (2013). The effect of extraction solvents, temperature and time on the composition and mass fraction of polyphenols in dalmatian wild sage (*Salvia officinalis* L.) extracts. FTB.

[55] Blažeković B., Vladimir-Knežević S., Brantner A., Bival S.M. (2010). Evaluation of antioxidant potential of *Lavandula x intermedia* Emeric ex Loisel. ’Budrovka’: A comparative study with *L. angustifolia* Mill. Molecules.

[56] Antony A., Farid M. (2022). Effect of temperatures on polyphenols during extraction. Appl. Sci..

[57] Prokopenko Y., Tsyvunin V., Shtrygol S., Georgiyants V. (2015). In vivo anticonvulsant activity of extracts and protopine from the *Fumaria schleicheri* herb. Sci. Pharm..

[58] Rojas-García A., Fernández-Ochoa Á., Cádiz-Gurrea M.L., Arráez-Román D., Segura-Carretero A. (2023). Neuroprotective effects of agri-food by-products rich in phenolic compounds. Nutrients.

[59] Adaszyńska-Skwirzyńska M., Dzięcioł M. (2017). Comparison of phenolic acids and flavonoids contents in various cultivars and parts of common lavender (*Lavandula angustifolia*) derived from Poland. Nat. Prod. Res..

[60] Gabriela S., Aonofriesei F., Simona L., Popescu A., Sirbu R. (2019). Study of phenolic compounds and antimicrobial activity of *Lavandula angustifolia* L. flowers macerates. Rev. Chim..

[61] Spigno G., Tramelli L., Dante Marco D.F. (2007). Effects of extraction time, temperature and solvent on concentration and antioxidant activity of grape marc phenolics. J. Food Eng..

[62] Onyebuchi C., Kavaz D. (2020). Effect of extraction temperature and solvent type on the bioactive potential of *Ocimum gratissimum* L. extracts. Sci. Rep..

[63] (2023). European Pharmacopeia 11.0.

[64] Bahadur S., Naurange T., Baghel P., Sahu M., Yadu K. (2020). Targeting the brain: Various approaches and science involved. Sci. Pharm. Sci..

[65] Volkova A., Proskurova I., Yevsieieva L. (2020). Study the attitude of the Ukrainian citizens to the environmental problems in the context of the theory of generations. Sci. Pharm. Sci..

[66] Fedotova M., Panfilova H., Hala L., Lebedyn A., Simonian L., Gerush O., Iurchenko G., Palamar A., Sholoiko N., Velia M. (2022). Evaluation of the state of pharmaceutical supply of patients with dementia with Alzheimer disease in Ukraine in accordance with international recommendations. Sci. Pharm. Sci..

[67] Mykhailenko O., Saidov N.B., Ivanauskas L., Georgiyants V. (2022). Model implementation of the legal regulation on medicinal plant cultivation for pharmaceutical purposes. Case study of *Crocus sativus* cultivation in Ukraine. Botanica.

